# State-wide situation analysis of availability of basic resuscitation devices and essential drugs in primary and secondary healthcare facilities in Cross River State, Nigeria

**DOI:** 10.11604/pamj.2022.42.225.35452

**Published:** 2022-07-21

**Authors:** Queeneth Ndukwe Kalu, Teresa Abang Edentekhe, Ogban Ezukwa Omoronyia, Bassey Etim Nakanda, Arit Ayi Archibong

**Affiliations:** 1 Department of Anaesthesia, University of Calabar, Calabar, Nigeria,; 2 Department of Community Medicine, University of Calabar, Calabar, Nigeria,; 3 Department of Anaesthesia, University of Calabar Teaching Hospital, Calabar, Nigeria,; 4 Department of Family and Emergency Medicine Faith Foundation Medical, Calabar, Nigeria

**Keywords:** Basic life support, devices, essential drugs, emergency, sudden death, Nigeria

## Abstract

**Introduction:**

Provision of Basic Life Support (BLS) to victims of cardiac arrest and other common causes of sudden death, is a key function of healthcare systems. Such life-saving service, which is lacking in many low- and middle-income countries (LMIC), is highly dependent on consistent availability of BLS devices and essential drugs. These devices are used to secure airway, deliver oxygen, gain intravenous access for infusions, provide cardiac defibrillation and monitor the cardiorespiratory systems. This study was aimed at evaluating the current state of availability of these devices and essential drugs in healthcare facilities in a developing country setting, within the context of urgent need to curb increasing burden of preventable sudden death.

**Methods:**

descriptive cross-sectional study design was employed to assess availability of each of the aforementioned subgroups of resuscitation devices and drugs, in each primary and secondary healthcare facility in all eighteen (18) Local Government Areas (LGAs) of Cross River State, in Southern Nigeria. Quantitative data was obtained using structured proforma, which was used to document presence and quantity of physically seen device(s) and drugs in each facility. The proportion of health facilities with presence of the devices and drugs, was compared between the three districts using chi-square test. P-value was set at 0.05.

**Results:**

two hundred and five (205) health care facilities across the eighteen (18) LGAs of Cross River State were assessed. Approximately one-tenth of health facilities had oropharyngeal airway (10.2%) and laryngoscope (9.3%). Only 5.4% and 3.9% had nasopharyngeal and endotracheal tubes, respectively. None of all of these airway devices was found in all health facilities within four LGAs (22.2%). The most commonly available breathing device was self-inflation bag (SIB), which was found in 51.7% of facilities. Seven LGAs (38.9%) had all of their health facilities not having either oxygen delivery devices, oxygen supply or both. Most health facilities had each of the IV access devices and infusion fluids, but only five facilities had automated external defibrillator (AED). Most health facilities had stethoscope (91.2%) and sphygmomanometer (72.2%), but only 15.1% and 9.3% had pulse oximeter and airway nebulizer, respectively. Less than one-fifth (18.5%) of facilities had atropine, and only 3.9% had amiodarone. Except for amiodarone, there was significantly higher proportion of health facilities that had each of the other essential drugs, in northern compared with other districts (p<0.05).

**Conclusion:**

devices and essential drugs required for provision of resuscitation are lacking in most health facilities in Cross River State. This situation significantly limits the health system’s capacity to save lives, especially during emergencies. The implications of these state-wide findings, as well as modalities and options for improvement in availability of these essential devices and drugs are discussed in this article.

## Introduction

Provision of Basic resuscitation to victims of cardiac arrest and other common causes of sudden death, is a key function of healthcare systems [1]. Such life-saving service, which is lacking in many low- and middle-income countries (LMIC), is highly dependent on consistent availability of BLS devices and essential drugs [2]. These devices are used to secure airway, deliver oxygen, gain intravenous access for infusions, provide cardiac defibrillation and monitor the cardiorespiratory systems [1]. Consistent availability of these devices and drugs, constitutes one of the four key requirements for sustainable provision and access to resuscitation services during events of sudden cardiac arrest in any setting [3]. The other three components are means of client or resuscitation service provider mobility (either from point of sudden cardiac arrest to where resuscitation may be provided, or of skilled personnel to point of sudden cardiac arrest), availability of skilled personnel and affordability of the services. Hence, in resource-limited settings, inadequate transportation networks, poor coverage of health insurance, general lack of basic health infrastructure and skilled personnel, may be key rationale for lack of effective basic and advanced life support services [3]. Unfortunately, there is paucity of research aimed at better understanding of these essential components, towards improvement in availability of BLS, at least in healthcare settings. Most studies on BLS in developing country settings, focus on effects of training interventions on skill development [4-7].

Training of skilled personnel in basic and advanced life support must go hand in hand with the availability of these devices and drugs especially in the face of scarce financial resources [3]. Yet, though it may generally be assumed that most health care facilities in developing countries lack infrastructure for cardio pulmonary resuscitation, there is lack of published literature on the real situation of institutional deficiency [5-7]. Hence, this study was aimed at on-the-spot situation analysis of availability of BLS devices and essential drugs, across the entire Cross River State, in Southern Nigeria, which may be considered to be a typical developing country setting. Findings will make significant contribution to lack of literature, as well as provide baseline for evidence-based interventions for improvement in at least one of the aforementioned components, required for sustainable availability of effective prevention and control of sudden deaths, within the context of high burden of the menace in the region [8,9].

## Methods

**Study design and setting:** descriptive cross-sectional study design was employed to assess availability of each of the aforementioned subgroups of BLS devices and drugs, in each primary and secondary healthcare facility in all eighteen (18) Local Government Areas (LGAs), distributed across the three senatorial districts of Cross River State, in Southern Nigeria. In Nigeria, each ward, as the simplest political unit within LGAs, has at least a Primary Health Center (PHC). Hence, the Primary Health Centers (PHCs) are typically established in each ward at creation of LGAs. Six (6) of the LGAs had been in existence since 1974, when the State was created out of the old South Eastern State. The remaining LGAs were created in 1988 when Akwa Ibom State was carved out of Cross River State. Hence, each LGA and therefore PHC, is at least 35 years old. Though PHCs are directly under the National Primary Healthcare Development Agency, direct oversight supervision is carried out by designated PHC coordinator in the LGA and professional nurse or Community Health Officer (CHO) at the facility. However, due to high degree of task-shifting amidst lack of doctors and nurses, most services at PHCs, including maternity and resuscitation during events of sudden deaths, are carried out by community health workers, comprising Community Health Extension Workers (CHEWs) and CHOs, who are typically much lower-skilled health workers. The secondary health facilities, comprising one general hospital in each LGA, proportionally have much more nurses and doctors, as well as less community health workers.

**Data collection and ethics:** quantitative data was obtained using structured proforma, which was used to document presence and quantity of physically seen BLS device(s) and drugs in each facility. Data collection was carried out for four (4) weeks beginning 10^th^ January, through 6th February, 2022. The health facility list, locations and focal persons, were obtained from the Cross River State Ministry of Health, Primary Healthcare Development Agency (PHCDA) and the PHC coordinators in each LGA. Trained research assistants were engaged for data collection for ten (10) consecutive days across the State, including facilities located in hard-to-reach rural settlements. Ethical approval for the study was obtained from the Cross River State Health Research Ethics Committee, while permission was obtained from the Director general PHCDA, the PHC coordinator in each LGA as well as heads of each facility before data collection.

**Instruments and data analysis:** structured proforma used for data collection comprised four sections. Sections 1, 2, 3 and 4 obtained information on physical presence of devices for securing airway/giving oxygen, intravenous access, breathing/circulation and essential drugs, respectively. Data was entered and analyzed using SPSS version 26.0 software. In each LGA, we computed the proportion of health facilities that had BLS devices or drugs of interest. We also compared the proportion across the 3 senatorial districts using Chi-squared test at a significance level of 0.05. Projection from prior census figures, was used to obtain population estimates for each LGA.

**Ethics approval and consent to participate:** ethical approval for the study was obtained from the Cross River State Research Ethics Committee.

## Results

**Figure 1:**
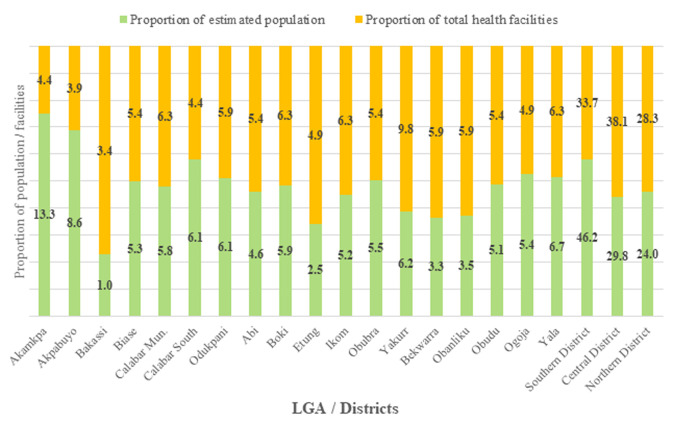
multiple bar chart showing proportion of estimated population and health facilities for each LGA and senatorial district

Two hundred and five (205) health care facilities across the eighteen (18) LGAs of Cross River State were assessed, comprising 69, 78 and 58, from southern, central and northern senatorial districts, respectively. Akamkpa was the most populated (419,705 people), while Yakurr had the highest number of health facilities (20) (Table 1). Akamkpa (13.3%) and the southern district (46.2%) had the highest proportion of estimated population, while Obanliku (3.5%), Bekwarra (3.3%) and Bakassi (1.0%) had low proportions (Figure 1). Yakurr (9.8%) and the central district (38.1%) had the highest proportion of health facilities, while Bakassi (3.4%) had the least proportion. Akamkpa and Bakassi had the highest and lowest degrees of disproportionately high estimated population compared with proportion of health facilities, respectively. Approximately one-tenth of health facilities had oropharyngeal tube (10.2%) and laryngoscope (9.3%). Only 5.4% and 3.9% had nasopharyngeal and endotracheal tubes, respectively (Table 2). None of all of these airway devices was found in all health facilities within four (22.2%) LGAs, comprising Akpabuyo, Boki, Etung and Obudu. Suction machine and resuscitation trolley were found in 28.8% and 27.3% of facilities, respectively. Except for Akpabuyo, all other LGAs had at least one health facility with suction machine. Also, except for Etung, all other LGAs had at least one health facility with resuscitation trolley.

**Table 1:** demographic description of study areas

Local Government Areas	Estimated population	Proportion of estimated population	Number of health facilities	Proportion of total health facilities
Akamkpa	419,705	13.27	9	4.39
Akpabuyo	272,262	8.61	8	3.90
Bakassi	31,641	1.00	7	3.41
Biase	168,113	5.32	11	5.37
Calabar Municipality	183,681	5.81	13	6.34
Calabar South	191,515	6.05	9	4.39
Odukpani	192,884	6.10	12	5.85
**Southern District sub-total**	1,459,801	46.15	69	33.66
Abi	144,317	4.56	11	5.37
Boki	186,611	5.90	13	6.34
Etung	80,036	2.53	10	4.88
Ikom	163,691	5.18	13	6.34
Obubra	172,543	5.46	11	5.37
Yakurr	196,271	6.21	20	9.76
**Central District sub-total**	943,469	29.83	78	38.05
Bekwarra	105,497	3.34	12	5.85
Obanliku	109,633	3.47	12	5.85
Obudu	161,457	5.10	11	5.37
Ogoja	171,574	5.42	10	4.88
Yala	211,557	6.69	13	6.34
**Northern District sub-total**	759,718	24.02	58	28.29
**TOTAL**	3,162,988		205	

**Table 2:** state-wide health facility assessment of health facilities with airway and oxygen devices

District/Local Govt. Area	Oroph. tube# viable facilities n (%)	Nasoph. tube# viable facilities n (%)	Endotr. tube# viable facilities n (%)	Suction. mchine# viable facilities n (%)	Soluble lubricant# viable facilities n (%)	Laryng. scope# viable facilities n (%)	Resus. trolley# viable facilities n (%)	Oxygen supply# viable facilities n (%)	O_2_ deliv devices# viable facilities n (%)	Self-infl. bag# viable facilities n (%)
**South District**	8 (11.6)	5 (7.2)	3 (4.3)	17 (24.6)	9 (13.0)	6 (8.7)	23 (33.3)	6 (8.7)	9 (13.0)	26 (37.7)
Akamkpa	1 (11.1)	0 (0)	0 (0)	2 (22.2)	1 (11.1)	1 (11.1)	4 (44.4)	1 (11.1)	2 (22.2)	1 (11.1)
Akpabuyo	0 (0)	0 (0)	0 (0)	0 (0)	0 (0)	0 (0)	2 (25)	0 (0)	1 (12.5)	3 (37.5)
Bakassi	1 (14.3)	1 (14.3)	1 (14.3)	1 (14.3)	1 (14.3)	0 (0)	1 (14.3)	0 (0)	1 (14.3)	2 (28.6)
Biase	5 (45.5)	2 (18.2)	1 (9.1)	5 (45.5)	2 (18.2)	1 (9.1)	4 (36.4)	1 (9.1)	2 (18.2)	7 (63.6)
Cal. Mun.	0 (0)	1 (7.7)	0 (0)	5 (38.5)	3 (23.1)	2 (15.4)	4 (30.8)	1 (7.7)	0 (0)	5 (38.5)
Cal. South	1 (11.1)	1 (11.1)	1 (11.1)	3 (33.3)	2 (22.2)	1 (11.1)	7 (77.8)	3 (33.3)	2 (22.2)	4 (44.4)
Odukpani	0 (0)	0 (0)	0 (0)	1 (8.3)	0 (0)	1 (8.3)	1 (8.3)	0 (0)	1 (8.3)	4 (33.3)
**Central District**	5 (6.4)	3 (3.8)	2 (2.6)	22 (28.2)	7 (9.0)	4 (5.1)	13 (16.7)	11 (14.1)	12 (15.4)	45 (57.7)
Abi	2 (18.2)	0 (0)	0 (0)	2 (18.2)	1 (9.1)	3 (27.3)	5 (45.5)	2 (18.2)	4 (36.4)	7 (63.6)
Boki	0 (0)	0 (0)	0 (0)	4 (30.8)	2 (15.4)	0 (0)	1 (7.7)	3 (23.1)	1 (7.7)	8 (61.5)
Etung	0 (0)	0 (0)	0 (0)	1 (10)	0 (0)	0 (0)	0 (0)	0 (0)	0 (0)	7 (70)
Ikom	1 (7.7)	1 (7.7)	1 (7.7)	7 (53.8)	1 (7.7)	1 (7.7)	1 (7.7)	3 (23.1)	4 (30.8)	7 (53.8)
Obubra	1 (9.1)	1 (9.1)	0 (0)	1 (9.1)	1 (9.1)	0 (0)	2 (18.2)	1 (9.1)	1 (9.1)	9 (81.8)
Yakurr	1 (5)	1 (5)	1 (5)	7 (35)	2 (10)	0 (0)	4 (20)	2 (10)	2 (10)	7 (35)
**North District**	8 (13.8)	3 (5.2)	3 (5.2)	20 (34.5)	13 (22.4)	9 (15.5)	20 (34.5)	3 (5.2)	7 (12.1)	35 (60.3)
Bekwarra	2 (16.7)	1 (8.3)	1 (8.3)	4 (33.3)	3 (25)	1 (8.3)	1 (8.3)	1 (8.3)	2 (16.7)	9 (75.0)
Obanliku	3 (25)	1 (8.3)	0 (0)	3 (25)	1 (8.3)	1 (8.3)	2 (16.7)	1 (8.3)	2 (16.7)	5 (41.7)
Obudu	0 (0)	0 (0)	0 (0)	3 (27.3)	0 (0)	1 (9.1)	1 (9.1)	1 (9.1)	1 (9.1)	5 (45.5)
Ogoja	1 (10)	1 (10)	1 (10)	3 (30)	3 (30)	2 (20)	7 (70)	0 (0)	2 (20)	9 (90)
Yala	2 (15.4)	0 (0)	1 (7.7)	7 (53.8)	2 (15.4)	4 (30.8)	9 (69.2)	0 (0)	0 (0)	7 (53.8)
**All LGAs/Districts**	21 (10.2)	11 (5.4)	8 (3.9)	59 (28.8)	25 (12.2)	19 (9.3)	56 (27.3)	20 (9.8)	28 (13.7)	106 (51.7)
**Chi-square comparison of proportions between districts**	0.33	0.66	0.72	0.47	0.08	0.12	0.03*	0.21	0.84	0.02*

* Statistically significant p-value

The most commonly available breathing device was self-inflation bag (SIB), which was found in 51.7% of facilities. Except for Akamkpa which had one, and Bakassi which had two, all other LGAs had at least three health facilities with SIB. Ogoja (90%), Obubra (81.8%) and Bekwarra (75.0%) had relatively higher proportion of health facilities with SIB. However, only 13.7% and 9.8% of health facilities had oxygen delivery devices and oxygen supply, respectively. Seven LGAs (38.9%), comprising Calabar Municipality, Akpabuyo, Bakassi, Odukpani, Etung, Ogoja and Yala had all of their health facilities not having either oxygen delivery devices, oxygen supply or both. There was higher proportion of health facilities with SIB in northern (60.3%), compared with central (57.7%) and southern (37.7%) districts (p<0.05). Also, resuscitation trolley was found in significantly lower proportion of health facilities in central (16.7%), compared with southern (33.3%) and northern (34.5%) districts (p<0.05). There was no significant difference in proportional distribution of other airway and breathing devices in health facilities comparing the districts (Table 2). Most health facilities had each of the IV access devices and infusion fluids (Table 3). These comprised IV cannula (75.6%), needles and syringes (92.7%), sharps container (92.7%), disposable gloves (86.3%), gowns and aprons (88.8%), IV fluids (86.3%) and drip stand (91.2%). However, only five facilities, located in Akamkpa, Akpabuyo, Bakassi, Odukpani and Yakurr had automated external defibrillator. Most facilities in central district except one in Yakurr LGA, and all facilities in northern district did not have AED device.

**Table 3:** state-wide health facility assessment of health facilities with IV access, infusion and AED devices

District/Local Govt. Area	IV Canulla# viable facilities (%)	Needles syringes# viable facilities (%)	Sharps container# viable facilities (%)	Disposable gloves# viable facilities (%)	Gowns and aprons # viable facilities (%)	IV fluids (NS/RL) # viable facilities (%)	Drip stand# viable facilities (%)	AED (defibrilator) #viable facilities (%)
**South District**	45 (65.2)	62 (89.9)	60 (87.0)	54 (78.3)	57 (82.6)	51 (73.9)	58 (84.1)	4 (5.8)
Akamkpa	6 (66.7)	8 (88.9)	7 (77.8)	6 (66.7)	5 (55.6)	7 (77.8)	8 (88.9)	1 (11.1)
Akpabuyo	4 (50.0)	6 (75.0)	7 (87.5)	5 (62.5)	8 (100.0)	4 (50)	6 (75)	1 (12.5)
Bakassi	3 (42.9)	7 (100)	7 (100)	3 (42.9)	3 (42.9)	3 (42.9)	3 (28.6)	1 (14.3)
Biase	9 (81.8)	9 (81.8)	7 (63.6)	10 (90.9)	10 (90.9)	10 (90.9)	11 (100)	0 (0)
Calabar Municipality	11 (34.6)	12 (92.3)	13 (100)	12 (92.3)	13 (100)	12 (92.3)	12 (92.3)	0 (0)
Calabar South	7 (77.8)	9 (100)	9 (100)	9 (100)	9 (100)	7 (77.8)	9 (100)	9 (100)
Odukpani	5 (41.7)	11 (91.7)	10 (83.3)	9 (75)	9 (75)	8 (66.7)	10 (83.3)	1 (8.3)
**Central District**	60 (76.9)	72 (92.3)	76 (97.4)	68 (87.2)	73 (93.6)	71 (91.0)	73 (93.6)	0 (0.0)
Abi	7 (63.6)	11 (100)	11 (100)	11 (100)	11 (100)	9 (81.8)	11 (100)	0 (0)
Boki	13 (100)	13 (100)	12 (92.3)	11 (84.6)	12 (92.3)	13 (100)	12 (92.3)	0 (0)
Etung	10 (100)	10 (100)	10 (100)	10 (100)	10 (100)	10 (100)	10 (100)	0 (0)
Ikom	8 (61.5)	11 (84.6)	12 (92.3)	13 (100)	11 (84.6)	13 (100)	12 (92.3)	0 (0)
Obubra	7 (63.6)	9 (81.8)	11 (100)	8 (72.7)	11 (100)	9 (81.8)	11 (100)	0 (0)
Yakurr	15 (75)	18 (90)	20 (100)	15 (75)	18 (90)	17 (85)	16 (80)	0 (0)
**North District**	50 (86.2)	56 (96.6)	54 (93.1)	55 (94.8)	52 (89.7)	55 (94.8)	56 (96.6)	1 (1.7)
Bekwarra	10 (83.3)	12 (100)	12 (100)	11 (91.7)	11 (91.7)	11 (91.7)	10 (83.3)	1 (8.3)
Obanliku	11 (91.7)	12 (100)	11 (91.7)	12 (100)	9 (75)	12 (100)	12 (100)	0 (0)
Obudu	8 (72.7)	11 (100)	10 (90.9)	11 (100)	11 (100)	10 (90.9)	11 (100)	0 (0)
Ogoja	8 (80)	8 (80)	8 (80)	9 (90)	9 (90)	9 (90)	10 (100)	0 (0)
Yala	13 (100)	13 (100)	13 (100)	12 (92.3)	12 (92.3)	13 (100)	13 (100)	0 (0)
**All LGAs/Districts**	15 (75.6)	190 (92.7)	190 (92.7)	177 (86.3)	18 (88.8)	177 (86.3)	187 (91.2)	5 (2.4)
**Chi-square comparison of proportions between districts**	0.02*	0.35	0.05	0.02*	0.11	0.00*	0.02*	0.07

* Statistically significant p-value

Most health facilities had stethoscope (91.2%) and sphygmomanometer (72.2%), but only 15.1% and 9.3% had pulse oximeter and airway nebulizer, respectively (Table 4). The proportion of health facilities with other devices for monitoring vital signs included thermometer (88.3%), glucometer (51.2%), urinary catheter (58.5%) and algorithm records (65.4%). Algorithm record was more commonly found in facilities in northern (77.6%) and central (71.8%) compared with southern (47.8%) districts (p<0.05). Concerning essential drugs for BLS, most health facilities had adrenaline (75.6%), diazepam (63.9%) and lignocaine (76.6%) (Table 5). Dextrose, calcium gluconate and aspirin were found in 39.5%, 37.1% and 34.6% of facilities, respectively. Less than one-fifth (18.5%) of facilities had atropine, and only 3.9% had amiodarone. Except for amiodarone, there was significantly higher proportion of health facilities that had each of the other essential drugs, in northern compared with other districts (p<0.05).

**Table 4:** state-wide health facility assessment of health facilities with airway, breathing, circulation monitoring devices

District/Local Govt. Area	Stethoscope# viable facilities n (%)	Pulse oximeter # viable facilities n (%)	BP Sphyg.# viable facilities n (%)	Airway nebulizer # viable facilities n (%)	Therm # viable facilities n (%)	Glucometer/strips # viable ffacilities n (%)	Urinary catheter # viable facilities n (%)	Multipar. monitor# viable facilities n (%)	Algorithm records # viable facilities n (%)
**South District**	58 (84.1)	15 (21.7)	38 (55.1)	7 (10.1)	57 (82.6)	33 (47.8)	32 (46.4)	4 (5.8)	33 (47.8)
Akamkpa	6 (66.7)	3 (33.3)	4 (44.4)	1 (11.1)	8 (88.9)	4 (44.4)	3 (33.3)	0 (0)	4 (44.4)
Akpabuyo	5 (62.5)	2 (25)	4 (50)	0 (0)	6 (75.0)	3 (37.5)	5 (62.5)	0 (0)	1 (12.5)
Bakassi	3 (42.9)	1 (14.3)	3 (42.9)	2 (28.6)	2 (28.6)	1 (14.3)	2 (28.6)	0 (0)	2 (28.6)
Biase	10 (90.9)	4 (36.4)	6 (54.5)	2 (18.2)	8 (72.7)	7 (63.6)	4 (36.4)	2 (18.2)	2 (18.2)
Cal Mun.	13 (100)	2 (15.4)	7 (53.8)	0 (0)	13 (100)	7 (53.8)	8 (61.5)	0 (0)	13 (100)
Cal South	9 (100)	2 (22.2)	5 (55.6)	2 (22.2)	1 (11.1)	6 (66.7)	6 (66.7)	2 (22.2)	6 (66.7)
Odukpani	12 (100)	1 (8.3)	9 (75)	0 (0)	12 (100)	5 (41.7)	4 (33.3)	0 (0)	5 (41.7)
**Central District**	74 (94.9)	10 (12.8)	68 (87.2)	8 (10.3)	68 (87.2)	37 (47.4)	43 (55.1)	6 (7.7)	56 (71.8)
Abi	11 (100)	3 (27.3)	8 (72.7)	0 (0)	11 (100)	4 (36.4)	7 (63.6)	1 (9.1)	10 (90.9)
Boki	12 (92.3)	3 (23.1)	11 (84.6)	4 (30.8)	10 (76.9)	9 (69.2)	6 (46.2)	1 (7.7)	11 (84.6)
Etung	9 (90)	0 (0)	10 (100)	0 (0)	10 (100)	6 (60)	5 (50)	1 (10)	10 (100)
Ikom	12 (92.3)	2 (15.4)	11 (84.6)	4 (30.8)	10 (76.9)	9 (69.2)	6 (46.2)	1 (7.7)	11 (84.6)
Obubra	1 (100)	0 (0)	10 (90.9)	0 (0)	10 (90.9)	4 (36.4)	7 (63.6)	0 (0)	9 (81.8)
Yakurr	19 (95)	2 (10)	18 (90)	1 (5)	18 (90)	8 (40)	12 (60)	2 (10)	5 (25)
**North District**	55 (94.8)	6 (10.3)	42 (72.4)	4 (6.9)	56 (96.6)	35 (60.3)	45 (77.6)	4 (6.9)	45 (77.6)
Bekwarra	11 (91.7)	1 (8.3)	12 (100)	1 (8.3)	11 (91.7)	7 (58.3)	6 (50.0)	2 (16.7)	11 (91.7)
Obanliku	11 (91.7)	2 (16.7)	12 (100)	1 (8.3)	12 (100)	7 (58.3)	11 (91.7)	1 (8.3)	11 (91.7)
Obudu	11 (100)	1 (9.1)	10 (90.9)	1 (9.1)	11 (100)	5 (45.5)	7 (63.6)	0 (0)	11 (100)
Ogoja	9 (90)	2 (20)	4 (40)	1 (10)	10 (100)	7 (70)	9 (90)	0 (0)	7 (70)
Yala	13 (100)	0 (0)	4 (30.8)	0 (0)	12 (92.3)	9 (69.2)	12 (92.3)	1 (7.7)	5 (38.5)
**All LGAs/Districts**	187 (91.2)	31 (15.1)	148 (72.2)	19 (9.3)	181 (88.3)	105 (51.2)	120 (58.5)	14 (6.8)	134 (65.4)
**Chi-square comparison of proportions between districts**	0.04*	0.16	0.00*	0.76	0.05	0.26	0.00*	0.90	0.00*

* Statistically significant p-value

**Table 5:** state-wide health facility assessment of available essential drugs

District/Local Govt. Area	Adrenaline# viable facilities (%)	Diazepam # viable facilities (%)	Atropine# viable facilities (%)	Dextrose # viable facilities (%)	Amiodarone# viable facilities (%)	Aspirin# viable facilities (%)	Ca Gluconate # vviable facilities (%)	Lignocaine # viable facilities (%)
**South District**	44 (63.8)	27 (39.1)	9 (13)	30 (43.5)	3 (4.3)	16 (23.2)	29 (42)	44 (63.8)
Akamkpa	5 (55.6)	5 (55.6)	2 (22.2)	0 (0)	0 (0)	2 (22.2)	2 (22.2)	3 (33.3)
Akpabuyo	4 (50)	3 (37.5)	0 (0)	2 (25)	0 (0)	1 (12.5)	2 (25)	4 (50)
Bakassi	2 (28.6)	2 (28.6)	1 (14.3)	1 (14.3)	0 (0)	2 (28.6)	3 (42.9)	3 (42.9)
Biase	7 (63.6)	5 (45.5)	3 (27.3)	6 (54.5)	1 (9.1)	2 (18.2)	5 (45.5)	10 (90.9)
Calabar Mun.	11 (84.6)	4 (30.8)	0 (100)	7 (53.8)	0 (0)	2 (15.4)	5 (38.5)	11 (84.6)
Calabar South	5 (55.6)	3 (33.3)	2 (22.2)	6 (66.7)	2 (22.2)	3 (33.3)	7 (77.8)	6 (66.7)
Odukpani	10 (83.3)	5 (41.7)	1 (8.3)	8 (66.7)	0 (0)	4 (33.3)	5 (41.7)	7 (58.3)
**Central District**	60 (76.9)	53 (67.9)	10 (12.8)	17 (21.8)	3 (3.8)	21 (26.9)	19 (24.4)	62 (79.5)
Abi	7 (63.6)	6 (54.5)	1 (9.1)	5 (45.5)	0 (0)	0 (0)	3 (27.3)	7 (63.6)
Boki	12 (92.3)	12 (92.3)	2 (15.4)	2 (15.4)	1 (7.7)	6 (46.2)	5 (38.5)	11 (84.6)
Etung	9 (90)	9 (90)	0 (0)	1 (10)	0 (0)	4 (40)	1 (10)	9 (90)
Ikom	10 (76.9)	9 (69.2)	2 (15.4)	1 (7.7)	0 (0)	4 (30.8)	3 (23.1)	12 (92.3)
Obubra	8 (72.7)	5 (45.5)	1 (9.1)	2 (18.2)	1 (9.1)	2 (18.2)	4 (36.4)	8 (72.7)
Yakurr	14 (70)	12 (60)	4 (20)	6 (30)	1 (5)	5 (25)	3 (15)	15 (75)
**North District**	51 (87.9)	51 (87.9)	19 (32.8)	34 (58.6)	2 (3.4)	34 (58.6)	28 (48.3)	51 (87.9)
Bekwarra	11 (91.7)	9 (75)	3 (25)	6 (50)	0 (0)	4 (33.3)	7 (58.3)	11 (91.7)
Obanliku	12 (100)	12 (100)	6 (50)	7 (58.3)	1 (8.3)	7 (58.3)	3 (25)	12 (100)
Obudu	8 (72.7)	11 (100)	3 (27.3)	2 (18.2)	0 (0)	4 (36.4)	4 (36.4)	8 (72.7)
Ogoja	8 (80)	7 (70)	3 (30)	7 (70)	1 (10)	8 (80)	7 (70)	8 (80)
Yala	12 (92.3)	12 (92.3)	4 (30.8)	12 (92.3)	0 (0)	11 (84.6)	7 (53.8)	12 (92.3)
**All LGAs/Districts**	155 (75.6)	131 (63.9)	38 (18.5)	81 (39.5)	8 (3.9)	71 (34.6)	76 (37.1)	157 (76.6)
**Chi-square comparison of proportions between districts**	0.01*	0.00*	0.00*	0.00*	0.97	0.00*	0.01*	0.00*

* Statistically significant p-value

## Discussion

This study revealed that many primary and secondary health care facilities in Cross River State had very few basic or advanced equipment for maintaining airway patency with some having none at all. The availability of self-inflating Bags (SIBs) for basic ventilation in many health facilities though grossly inadequate was an improvement in comparison to airway devices. Oxygen was a very scarce commodity though relatively more available in the northern part of the State and in some facilities where oxygen was available, there were no oxygen therapy devices. Among the resuscitation drugs, adrenaline was available in most facilities whereas atropine and amiodarone were mostly absent. Conversely, injection needles, consumables for injection and infusions were available in most facilities. Monitoring equipment such as stethoscope and sphygmomanometers were more readily found than pulse oximeters. Majority of the facilities did not have automated external defibrillators and this situation spanned through the three senatorial districts of the State. The low possession of basic resuscitation adjuncts in many of the primary and secondary healthcare facilities in Cross River State is worrisome. Maintenance of a patent airway is critical to the survival of every unconscious patient or victim. This is because in the unconscious state, the victim loses all protective reflexes and the tongue commonly falls backwards to occlude the hypopharynx. Apart from the head tilt, jaw thrust and chin lift maneuvres, the simplest supraglottic airway device is the oropharyngeal airway (OPA). In this study, it was available in only 10% of the primary and secondary health facilities. The significance of this finding is that in the vast majority of health facilities across the State, unconscious victims rushed in, stand a high risk of death from asphyxia. A similar finding was recorded in a study in Botswana where availability of items for airway and breathing ranged from 9.2% to 24.1% [8]. The absence of nasopharyngeal airway devices was also reported in a study in Ethiopia [9]. Nasopharyngeal airways (NPA) are useful in maintaining airway patency even in semiconscious patients unlike the OPA which should be used in the deeply unconscious. The near absence of NPA in our facilities may be a reflection of a lack of knowledge of its use among these cadre of healthcare providers. It could also be due to the general poor funding and inadequate equipment in health facilities in LMIC. This is however, in contrast to a study in Namibia also an African country where an audit of emergency trolleys in state owned hospitals revealed 75% presence of the oropharyngeal airway [10].

The scarcity of the endotracheal tube is understandable since its insertion requires a higher skill set from anaesthetists, intensivists or trained emergency physicians and paramedics. Interestingly in the Namibian study, they reported the presence of endotracheal tubes, laryngoscopes and introducers in all the hospitals visited. However, while our study was a total enumeration of primary and secondary care facilities, the Namibian study used convenience sampling and the hospitals chosen were the largest in the region. A study of resuscitation capacity of tertiary and referral centres in Nigeria revealed greater availability of both airway and breathing equipment across the country [11]. As much as basic life support entails the use of little or no equipment, when a patient is brought to the health facility, it is expected that airway adjuncts should be available to maintain airway patency. This should include the supraglotic airway devices such as the oropharyngeal airway, the nasopharyngeal and laryngeal mask airway. Where there is skilled manpower, infraglottic airway devices such as the endotracheal tube, cricothyroidotomy and tracheostomy tubes should be available to rescue the airway in life threatening conditions. It is possible that these health facilities rarely manage life threatening airway emergencies and staff may not be proficient in the use of such equipment. On the other hand, other researchers have reported that apart from immunization services, the populace prefer accessing medical care from tertiary hospitals in urban areas and alternative medicine practitioners in the rural communities [12-14]. This study did not examine the case records in these facilities but it is common knowledge that primary health centres (PHCs) in Nigeria are actively involved with immunization of childhood diseases and routine Maternal and child health services. Currently, primary and secondary care facilities are involved in the COVID-19 vaccination exercise. Though rare, sudden collapse following immunization can occur [15,16]. COVID-19 immunization has been associated with increased incidence of myocarditis and pericarditis which are potentially fatal [15,17]. Facilities involved in immunization must therefore be in a state of readiness for resuscitation by offering both basic and advanced life support.

The absence of suction machines in over 70% of the facilities is also an unacceptable finding. This is a far cry from studies in Kano and Akwa Ibom States also in Nigeria where suction machine was present in 73% of the facilities [18,19]. It can be argued that these were questionnaire based studies not involving facility visits and verifications unlike ours. Most suction machines are electrically powered and erratic electricity supply may be a cause of non-procurement of such suction machines. There are however manually operated suction machines which should be available as alternatives in every emergency room where power supply poses a challenge. With the primary health care under one roof programme, PHCs are situated in every ward of the community and accessible within 5km [20-22]. They ought to be the first responders to emergencies. It is not clear how patients can be protected from regurgitation and aspiration where suction is needed in the current situation. The absence of such basic equipment will deprive patients of immediate response, delay emergency care and lead to unnecessary referrals with consequent overcrowding of tertiary care facilities. The cadre of staff particularly in our PHCs may be contributory to this state of inadequacies. Most PHCs are managed by Community health workers and community health extension workers. Physician led practices are known to have greater impact including advocacy for procurement of new equipment among other things [23,24].

The study revealed that 51.7% of the facilities possessed self-inflating bags (SIBs) commonly called Ambu bags. It is an improvement on the findings of a study in Akwa Ibom State also in Nigeria which revealed the presence of neonatal resuscitation SIBs in only 11.5% of the facilities assessed [18]. Self-inflating bags were found in most resuscitation trolleys audited in Namibia unlike Botswana where several study locations lacked SIBs [10,25]. While our finding revealed a significant increase in number of SIBs compared to the airway devices, it is still a far cry from the ideal. Every healthcare facility should not just possess the self-inflating bags but have various appropriate sizes for neonates, infants, children and adults. The relatively higher presence of SIBs implies that Healthcare Workers (HCWs) are more familiar with its use. It may be attributable to the Helping Babies Breath (HBB) aspect of Maternal and child health programme efforts of both the Federal government and the Paediatric Association of Nigeria [26]. This initiative requires that appropriate sizes of self-inflating bags be available in every facility. This study has obviously revealed that this is not the case currently. Many neonatal cardiac arrests are of respiratory aetiology. This piece of equipment must be available in different sizes for use when indicated to reduce not just neonatal but also maternal morbidity and mortality. Our findings underscore the need for emphasis on training to ensure an open airway before breathing during cardiopulmonary resuscitation (CPR) training programs as part of readiness for resuscitation in primary and secondary healthcare facilities.

Oxygen is one of the most used emergency drugs in developed countries. On the contrary, LMIC struggle to ensure availability of this essential resuscitation commodity. In this study, availability of oxygen and oxygen therapy devices was not impressive in the Southern part of the State which is close to the State Capital and tertiary hospitals compared to the central part of the State. We attributed this finding to the nearness to these referral centres. Studies have shown that patients in developing countries often bypass the primary and secondary care centres in preference for the tertiary facilities where they are sure of specialized care [12-14]. It is possible that if Specialist Family physicians are engaged to lead the services rendered in these peripheral centres, there could be a reversal of this trend. The revelation that patients requiring oxygen therapy cannot have access to it in many primary and secondary healthcare facilities poses a great threat to our medical services. Clearly, it will lead to preventable deaths and a worsening of our health indices including increased maternal and infant mortality if the situation is not corrected. The Kano study revealed the presence of Oxygen in 33.3% and 78.4% facilities possessed oxygen concentrators [19]. The availability of oxygen concentrators is an excellent alternative to oxygen cylinders in the absence of oxygen plants where these cylinders can be readily refilled. However, it requires electricity which is another major challenge in LMIC. Certain devices, such as intravenous infusion set, fluids, aprons, stethoscope and sphygmomanometer, were more readily available, compared with pulse oximeter, airway nebulizer. This is similar to findings in other studies which may indicate the familiarity with these monitoring devices [27-30]. The other rationale for this finding may be that unlike scarce devices, more readily available ones may be serving multiple purposes, therefore leading to their prioritization by healthcare managers, due to potential perception of their being more cost-efficient [29]. For instance, stethoscope is essentially utilized regularly by nurses and doctors in virtually all aspects in the spectrum of care provision, making it better prioritize than pulse oximeter which may be required only during emergency or close monitoring situations. Though availability of these devices should precede training of healthcare workers on how to use them, fear of possible eventual redundant or disuse status, may discourage health managers from procuring them. Scarcity of BLS devices and essential drugs, also reflects poor budgetary allocation to the healthcare sector in tune with the Abuja Declaration in 2000, and lack of willingness to upgrade the health infrastructure by the government of Nigeria towards attainment of Sustainable Development Goals [30,31]. By implication, healthcare workers in facilities that lack BLS devices and drugs, will continue to be deprived of the knowledge and skills for provision of full range of CPR in resource-limited settings including Nigeria. Persistence of this facility-based deprivation has contributed to increase in proportion of areas that are underserved regarding prevention and management of sudden deaths.

There was an appalling lack of defibrillators in over 70% of the local government areas. Most adult sudden cardiac arrests (SCA) are cardiovascular in origin and where there is ventricular fibrillation or ventricular tachycardia, defibrillation leading to successful resuscitation has been reported in up to 70% of cases [32]. Basic life support is therefore a holding procedure for defibrillation. The shorter the interval from arrest to defibrillation, the better the prognosis. Previous studies have shown that the best resuscitation outcomes occur if defibrillation occurs within 3 minutes and chances of successful resuscitation drops by 7-10% for every minute of delay in defibrillation thereafter [33]. Our findings indicate that the likelihood of any victim of SCA receiving this life saving intervention is extremely slim. This is worsened by the near absence of prehospital emergency medical services in most LMIC including many States in Nigeria. A study by United States Agency for International Development (USAID) indicated the distance from a basic obstetric care centre to a comprehensive centre as being up to 162 minutes in some cases [34]. In an event of SCA, even where there is bystander CPR, any willing rescuer would become exhausted with consequent ineffective chest compressions by that length of time. It is for this reason that drone delivery of automated external defibrillators (AEDs) has been introduced and public access defibrillators are recommended to be located in sports facilities, offices, cinemas, schools, markets in addition to hospitals [35,36].

Medications available for resuscitation were variable in this study. While adrenaline was present in 75.6%, amiodarone was found in barely 3.9% facilities. Similar variabilities were seen in other similar studies [10,25]. There is need for a standardized emergency drug trolley which should be regularly audited. Clearly as we build capacity on basic life support using adjuncts, we should gradually train on advanced life support as well as make HCWs familiar with resuscitation medications including when and how to use them. Skilled family physician led practices will likely attract the clientele, improve confidence in the primary and secondary facilities and reposition them for the purpose they were set up as the nearest health facilities to the people. This study found that the situation of unavailability of resuscitation devices and drugs, was significantly worse in some LGAs and senatorial districts. Availability may be highly dependent on forces of demand and supply of these essential items within the study area. In other words, settings with poorer situation of non-availability, may be a reflection of poor uptake or demand for resuscitation services, which in turn does not justify supply and stocking of the items at these facilities [10]. Poor uptake of CPR services, may also be due to possible prior knowledge of paucity of the requisite devices and skills in these facilities by residents in the area [10]. Yet, lack of stocking of BLS devices may be due to poor level of ability and willingness to pay for the services, especially in view of prevalent out-of-pocket mode of health expenditure amidst high burden of unemployment or underemployment in Nigeria [20,21]. This suggest that clients in need of cardiopulmonary resuscitation in these areas, may either deteriorate and possibly die, seek help in much further facilities with relatively more devices for CPR amidst life-threatening delays, or seek nearby help from quack local or traditional practitioners. Hence, within generally abysmal situation of unavailability of BLS devices statewide, there is perhaps vicious health inequality cycle of more severe lack in some areas and facilities, which are therefore potentially inactive for provision of CPR services, as well as possible burn out stress in facilities with more devices and drugs to provide the services [18,37]. Unfortunately, most BLS devices and drugs are not listed in the Essential Drug Revolving Fund system for ensuring sustainable availability [19,37].

The strength of this study is that it was a total enumeration of all the primary and secondary health centers in the State. This design provides a diverse representation from all the centres to permit inferences as to the current state of emergency services in Cross River State. The limitation is that a few of the health facilities were affected by the “END SARS” protest and were no longer functional. It is most unlikely that the findings in these negligible few facilities would have altered the result of this work. There is a slim chance that health workers might have assumed under-declaration of their equipment may attract funding or additional equipment. This assumption cannot however be ascertained.

## Conclusion

Devices and essential drugs required for provision of resuscitation is lacking in most health facilities in Cross River State. This situation significantly limits the health system´s capacity to save lives, especially during emergencies. The implications of these state-wide findings is that Cross River State like most others in LMIC is in a situation of unreadiness to manage cardiac arrests and other life threatening emergencies. The options for improvement include procurement and periodic monitoring using checklists to ensure the availability of the essential devices and drugs discussed in this article. There is an urgent need to ensure readiness for resuscitation services, through adequate stocking at health facilities and establishment of a mechanism for continuous capacity building of healthcare workers. This may require better understanding of the challenges of effective implementation of the drug revolving fund established at the Bamako Initiative, including updating list with inclusion of essential drugs used for resuscitation. Additionally, it is necessary to establish linkages among health facilities, for more effective coordination towards prevention of out-of-stock syndrome and expiry of medications.

**Funding:** this research work was supported by the TETFUND grant.

### What is known about this topic

- Availability of essential drugs and devices are key for effective provision of resuscitation services in healthcare systems and settings;
- In most LMICs, the few healthcare workers that have been trained on BLS, are unable to consistently and effectively provide resuscitation services due to lack of required devices and essential drugs.

### What this study adds

- This study bridges the knowledge gap on current state of availability of resuscitation devices and essential drugs for each LGAs and districts in the entire Cross River State;
- Study findings also provide baseline for evidence-based interventions for improvement in availability of resuscitation devices and essential drugs in the study setting.
